# Common Fibular Nerve Palsy in a Cyclist after Bariatric Surgery – Case Report

**DOI:** 10.1055/s-0042-1757964

**Published:** 2024-12-27

**Authors:** João Carlos Nakamoto, Bernardo Figueira Althoff, Ricardo Boso Escudero, Mauro Cesar Mattos e Dinato

**Affiliations:** 1 Instituto Vita, São Paulo, São Paulo, Brasil; 2 Hospital de Clínicas, Universidade Estadual de Campinas (Unicamp), Campinas, São Paulo, Brasil

**Keywords:** bariatric surgery, cycling, exercise, peroneal neuropathies, weight loss

## Abstract

Common fibular nerve (CFN) palsy is the most common mononeuropathy in the lower limb, and several etiologies are described. The CFN is the minor and lateral division of the sciatic nerve; it originates in the lumbar sacral division, and many risks of compression have been described: the behavior of crossing and squatting legs, extra and intraneural compressions, local trauma, and weight loss have been increasingly reported as important and noteworthy causes. The treatment is based on the severity of the nerve condition. In cases in which neurological impairment persists, surgical decompression is indicated. In cases of atraumatic palsy, compression of the fibular neck is the most important cause. The present is the report of a case of a 39-years-old male amateur cyclist who had undergone bariatric surgery and lost more than 30% of his initial body mass. Eleven months after the surgery, he performed a strenuous cycling session and evolved with paresthesia in dorsal left foot and dorsiflexion impairment. The electromyographic examination showed CFN palsy. The patient was submitted to surgical nerve decompression, with good results in ten months of follow-up. Strenuous physical activity after bariatric surgery with substantial weight loss can evolve with CFN palsy. This etiology should be considered in the clinical reasoning in such clinical presentation.

## Introduction

Common fibular nerve (CFN) palsy is one of the most common mononeuropathies in the body, the most common in the lower limbs, and several etiologies have been described. 1 When considered a non-traumatic lesion, compressions remain the most common cause. There are several risk factors for fibular compression: tumors, position of the legs, muscular edema and small hematoma on asthenic athletes, fibrous bands, changes in the bones, iatrogenic conditions, and others. 2 3 The association of rapid weight loss with prolonged physical exercise as a cause of CFN palsy has not been well reported. 4 We herein described a case of an amateur cyclist who experienced foot drop after a bariatric surgery.

## Case Report

The present case report was approved by the institutional ethics committee, and the patient signed the free and informed consent form.

A 39-year-old man weighing 123.80 Kg and 1.80 m tall (body mass index: 38.2Kg/m ^2^ ; body surface area: 2.404m ^2^ ), classified grade-II obese, 5 had been submitted to a bariatric surgery through the Santoro method (vertical gastroplasty). 6 He had only one kidney that had been transplanted four years before the bariatric surgery.

**Fig. 1:**
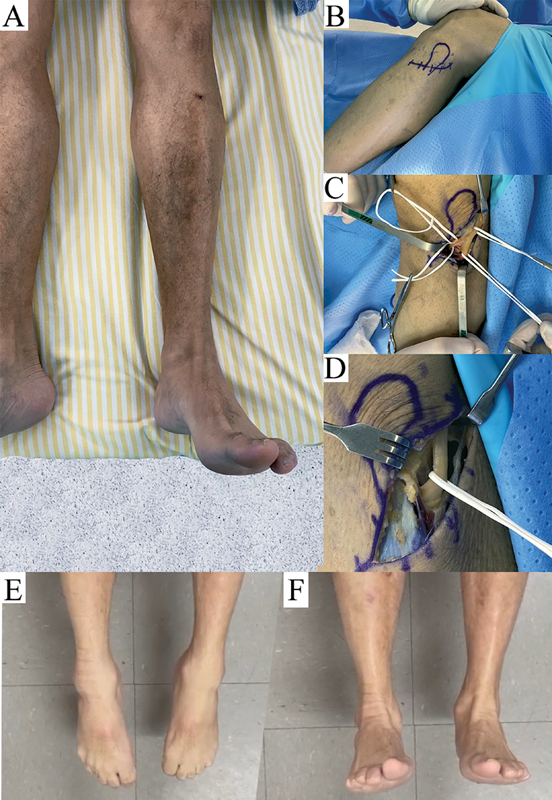
( **A** ) The maximum extension of the left foot shows the disability twenty days after the onset of symptoms. ( **B** ) Planning and ( **C,D** ) surgical decompression of the CFN. ( **E,F** ) Clinical evidence of complete recovery of the extension and full range of motion of the left feet 24 months after the surgery.

Two weeks after the bariatric surgery, the patient lost 14.3 Kg (11.6% of his initial weight). After 1 month, his weight was of 103.6 Kg (a loss of 16.6% of his initial weight). Eight months and a half after the surgery, he weighed 84 Kg (loss of 39.4 Kg and of 32.1% of his initial weight). During this period, the patient started to practice amateur long-distance cycling. Eleven months after the bariatric surgery, after a strenuous 70-Km ride in a straight route, he experienced paresthesia in anterior region of the distal leg and in the dorsal part of the foot. The next day, he rode another 45 Km downhill, and the day after that, he woke up feeling his left foot drop and loss in extension force ( Fig. 1A ).

He sought care two weeks after symptom onset. The physical examination did not show muscular atrophy, but showed an M2 in the anterior tibialis and paresthesia in the region of the CFN. An electroneuromyographic study was performed 20 days after the initial clinical symptoms, and it showed subacute sensory mononeuropathy and motor demyelinating neuropathy that was initially moderate to severe (with presence of conduction block and secondary degenerative axonal sign) at the level of the head of the fibula; thus, the diagnosis was confirmed. A metabolic study was performed, but the hemogram and the levels of B-complex vitamins were completely normal. Decompression was indicated and performed 15 months after bariatric surgery, 4 months after the onset of the neurological symptoms.

The surgery was performed with the patient under general anesthesia, using pneumatic tourniquets on the thigh with 340 mmHg. A 6-cm incision was made over the fibular neck ( Fig. 1B ) and, after a careful dissection, the compression of the CFN was revealed in the osteofibrous band of the origin of the long peroneal muscle. The nerve featured an hourglass with aspects of chronic compressive neuropathy. The fibrous band was released throughout the course of the nerve course, and a careful external neurolysis was performed with a 3.2 magnifying glass ( Fig. 1C and 1D ).

The anterior sensitivity in the distal leg and dorsal region of the left foot was partially recovered immediately after surgery. During the follow-up, there was a gradual improvement in the strength of the anterior muscles on physical examination. Seven days after the surgery, the patient experienced subsidence of the pain. After 10 weeks, the clinical examination revealed total recovery of strength and sensitivity, which is illustrated in photos taken 24 months postoperatively ( Fig. 1E and 1F ).

## Discussion

The CFN is the minor and lateral division of the sciatic nerve, and it originates in the lumbar sacral division (L4 to S2, posterior division). In the popliteal fossa, sciatic nerve divides into the CFN and the tibial nerve. The CFN crosses the lateral head of the gastrocnemius muscle and, as it courses to the head of the fibula, it emerges more subcutaneously and divides into superficial and deeper peroneal nerves. The former innervate the lateral muscle of the leg and provide sensitivity to the dorsal side of the feet, except to the first interdigital space. The latter innervate the muscles of the anterior compartment of the leg and provide sensitivity to the first space of the interdigital web. 1 On its course to the peroneal neck, the CFN takes the path of the peroneal tunnel, an osteofibrous band located at the origin of the long peroneal muscle that it is a common site of entrapment. 4

Several risk factors for compression of the CFN have been described in the literature: behavior of prolonged crossing and squatting of the legs, intra- and extraneural compressive masses, recent anesthesia, surgery and prolonged hospitalization, diabetes, and metabolic alterations such as malnutrition and vitamin complex deficiency, 4 a usual finding in obese patients, for 20% to 30% of them have nutritional disorders that may worsen after surgery. 5

Weight loss has been reported as a habitual cause of CFN neuropathy. The frequency of neurological complications among bariatric patients has been reported to range from 1.3% to 4.6% of the cases. 6 Some pathological causes have been described: loss of the protection of the subcutaneous tissue, which causes compression of the nerve against the hard structure of the bone, an immune mechanism of inflammatory infiltration into the microstructure of the nerve, such as cachexia, and the nutritional deficiencies. 7

Conversely, in athletic patients, the CFN is the nerve most affected by traumatic lesions, and blunt trauma is the most common mechanism. On the other hand, traction injuries, although less common, could be more severe. 8 Few sports have been associated with non-traumatic CFN palsy, such as chronic exertional compartment syndrome. In a study involving patients who were runners, Peck et al. 9 reported that a repetitive movement combining plantar flexion and inversion while running downhill or on uneven surfaces could cause a distension in the fibular head of the CFN. 9 The case herein reported is that of an amateur cyclist with a CFN lesion that may have worsened after extreme weight loss and a strenuous leg exercise, which was an unusual presentation of CFN palsy. Strenuous exercise can cause microtrauma in the muscle belly, which results in local edema and small hematomas, which may be another cause of compression due to physical exercise. 8

The treatment is based on the cause and severity of the nerve palsy. When the symptoms are intermittent and it is possible to make changes regarding lifestyle, a conservative treatment should be administered. Changing behavior in terms of position of the legs, sleep, exercises, posture, protecting the prominent fibular head and rehabilitation to stretch the contralateral muscle groups, and progressive strengthening of the dorsiflexors are some practices that must be performed. When any traumatic lesion or full motor or sensory function is lost, or there are no signs of improvement within 6 weeks to 3 months of the conservative treatment, a surgical approach and microneurolysis of the CFN are indicated. Good outcomes have been reported after neurolysis surgery, with 3% of cases of some motor or sensitive deficit. 2 9 10

Palsy of the CFN has countless causes, both traumatic and non-traumatic. However, weight loss followed by bariatric surgery and strenuous sports may be a possible etiology of CFN palsy.

## References

[1] Marciniak C Fibular (peroneal) neuropathy: electrodiagnostic features and clinical correlates Phys Med Rehabil Clin N Am 2013 24 01 121 137 23177035 10.1016/j.pmr.2012.08.016

[2] Poage C Roth C Scott B Peroneal nerve palsy: evaluation and management J Am Acad Orthop Surg 2016 24 01 1 10 26700629 10.5435/JAAOS-D-14-00420

[3] Fares M Y Dimassi Z Fares J Musharrafieh U Peroneal neuropathy and bariatric surgery: untying the knot Int J Neurosci 2020 130 04 417 423 31735096 10.1080/00207454.2019.1694926

[4] Katirji B Peroneal neuropathy Neurol Clin 1999 17 03 567 591, vii 10393754 10.1016/s0733-8619(05)70153-5

[5] Baima J Krivickas L Evaluation and treatment of peroneal neuropathy Curr Rev Musculoskelet Med 2008 1 02 147 153 19468889 10.1007/s12178-008-9023-6 PMC2684217

[6] Landais A Neurological complications of bariatric surgery Obes Surg 2014 24 10 1800 1807 25060718 10.1007/s11695-014-1376-x

[7] Thaisetthawatkul P Collazo-Clavell M L Sarr M G Norell J E Dyck P J A controlled study of peripheral neuropathy after bariatric surgery Neurology 2004 63 08 1462 1470 15505166 10.1212/01.wnl.0000142038.43946.06

[8] Meadows J R Finnoff J T Lower extremity nerve entrapments in athletes Curr Sports Med Rep 2014 13 05 299 306 25211617 10.1249/JSR.0000000000000083

[9] Peck E Finnoff J T Smith J Neuropathies in runners Clin Sports Med 2010 29 03 437 457 20610032 10.1016/j.csm.2010.03.002

[10] Kim D H Murovic J A Tiel R L Kline D G Management and outcomes in 318 operative common peroneal nerve lesions at the Louisiana State University Health Sciences Center Neurosurgery 2004 54 06 1421 1428, discussion 1428–1429 15157299 10.1227/01.neu.0000124752.40412.03

