# Supplementary material for: Common Fibular Nerve Palsy in a Cyclist after Bariatric Surgery – Case Report
Source: Rev Bras Ortop (Sao Paulo). 2024 Dec 27;59(Suppl 2):e176–9. doi: 10.1055/s-0042-1757964 (PMC11679703; doi:10.1055/s-0042-1757964)
Supplement: Related texts

# Research article: Paralisia do nervo fibular comum em um ciclista após cirurgia bariátrica – Relato de caso

**Keywords:** ciclismo, cirurgia bariátrica, exercício, neuropatias fibulares, perda de peso

## Abstract

A paralisia do nervo fibular comum (NFC) é a mononeuropatia mais comum no membro inferior, e várias etiologias são descritas. O NFC é a divisão menor e lateral do nervo ciático; ele se origina da divisão sacral lombar, e muitos riscos de compressão foram descritos: o comportamento de cruzar e agachar as pernas, compressões extra e intraneural, traumas locais e perda de peso vêm aparecendo como causas importantes de serem lembradas. O tratamento é baseado na gravidade da condição do nervo. No caso de os prejuízos neurológicos serem persistentes, a descompressão cirúrgica é indicada. Quando se trata de uma paralisia não traumática, a compressão do pescoço fibular é a causa mais importante. Este artigo apresenta o caso de um ciclista amador de 39 anos que foi submetido a cirurgia bariátrica e perdeu mais de 30% da massa corporal inicial. Onze meses após a cirurgia, ele fez uma sessão de exercício extenuante na bicicleta e evoluiu com parestesia no pé esquerdo dorsal e comprometimento da dorsiflexão. O exame eletromiográfico mostrou paralisia do NFC. Ele foi submetido a descompressão cirúrgica do nervo, com bons resultados até dez meses de seguimento. Atividade física extenuante após cirurgia bariátrica com perda substancial de peso pode evoluir com paralisia de NFC. Esta etiologia deve ser considerada no raciocínio clínico em tal apresentação clínica.

## Introdução

A paralisia do nervo fibular comum (NFC) é uma das mononeuropatias mais comuns no corpo, a mais comum em membros inferiores, e várias etiologias são descritas. 1 Quando considerada uma lesão não traumática, as compressões permanecem as causas mais comuns. Há vários fatores de risco para a compressão fibular: tumores, posicionamento das pernas, edema muscular e hematoma pequeno em atletas astênicos, bandas fibrosas, alterações nos ossos, condição iatrogênica, entre outros. 2 3 A associação da rápida perda de peso com exercícios físicos prolongados como causa da paralisia da NFC não é muito bem relatada. 4 Descrevemos um caso de um ciclista amador que sentia o pé cair após uma cirurgia bariátrica.

## Relato de Caso

Este relato de caso foi aprovado pelo Comitê de Ética institucional, e o paciente assinou o termo de consentimento livre e esclarecido.

Um homem de 39 anos, com 123,80 Kg de peso e 1,80 m de altura (índice de massa corporal: 38,2 kg/m ^2^ ; área da superfície corporal: 2,404 m ^2^ ), classificado como obeso de grau II, 5 foi submetido a uma cirurgia bariátrica pelo método Santoro (gastroplastia vertical). 6 Ele tinha apenas um rim, que fora transplantado quatro anos antes da cirurgia bariátrica.

Após 2 semanas da cirurgia bariátrica, o paciente perdeu 14,3 Kg (11,6% do peso inicial). Após um mês, seu peso era de 103,6 Kg (perda de 16,6% do peso inicial). Oito meses e meio após a cirurgia, ele estava com 84 Kg (havia perdido 39,4 Kg e 32,1% do peso inicial). Durante esse período, o paciente começou a praticar ciclismo amador de longas distâncias. Onze meses depois da cirurgia bariátrica, após um percurso extenuante de 70 Km em pista reta, ele percebeu parestesia na região anterior da perna distal e na parte dorsal do pé. No dia seguinte, ele percorreu mais 45 km em uma pista de descida, e no outro dia, acordou sentindo seu pé esquerdo cair e perda de força de extensão ( Fig. 1A ).

**Fig. 1:** ( **A** ) A extensão máxima do pé esquerdo mostra a incapacidade 20 dias após o início dos sintomas. ( **B** ) Planejamento e ( **C,D** ) descompressão cirúrgica do NFC. ( **E,F** ) Evidência clínica da extensão completa da recuperação e amplitude total do movimento dos pés esquerdos 24 meses após a cirurgia.

Ele procurou atendimento depois de duas semanas após o início dos sintomas. O exame físico revelou que não havia atrofia muscular, mas mostrou um M2 no músculo tibial anterior e parestesia em na área do NFC. Um estudo de eletroneuromiografia foi realizado 20 dias após os sintomas clínicos iniciais, e revelou uma mononeuropatia sensorial subaguda e desmielinização motora inicialmente moderada a grave do NFC (com presença de bloco de condução e sinal axonal degenerativo secundário) no nível da cabeça da fíbula; assim, o diagnóstico foi confirmado. Fez-se uma investigação metabólica, mas o hemograma e os níveis das vitaminas do complexo B estavam totalmente normais. A descompressão foi indicada e realizada 15 meses após a cirurgia bariátrica, 4 meses após o surgimento dos sintomas neurológicos.

A cirurgia foi realizada com o paciente sob anestesia geral, utilizando torniquetes pneumáticos na coxa com 340 mmHg. Fez-se uma incisão de 6 cm sobre o colo fibular ( Fig. 1B ) e, após uma dissecção cuidadosa, a compressão da NFC foi revelada na banda osteofibrosa da origem do músculo fibular longo. O nervo apresentava uma ampulheta com aspectos de neuropatia compressiva crônica. A banda fibrosa foi liberada ao longo do curso nervoso, e uma cuidadosa neurólise externa foi feita com uma lupa 3.2 ( Fig. 1C e 1D ).

A sensibilidade anterior da perna distal e da região dorsal do pé esquerdo foi parcialmente recuperada imediatamente após a cirurgia. Durante o acompanhamento, a força muscular anterior foi gradualmente melhorando no exame físico. Sete dias após a cirurgia, o paciente apresentou a subsidência da dor. Após 10 semanas, o exame clínico demonstrou recuperação total da força e da sensibilidade, ilustrada com fotos de 24 meses de pós-operatório ( Fig. 1E e 1F ).

## Discussão

O NFC é a divisão menor e lateral do nervo ciático, e tem a origem da divisão sacral lombar (L4 a S2, divisão posterior). Na fossa poplítea, o nervo ciático se divide em NFC e nervo tibial. O NFC cruza a cabeça lateral do músculo gastrocnêmico; assim que passa para a cabeça da fíbula, emerge de forma mais subcutânea, e se divide em nervos fibulares superficiais e mais profundos. O primeiro inerva o músculo lateral da perna e proporciona sensibilidade ao dorso dos pés, exceto ao primeiro espaço interdigital. O segundo inerva os músculos do compartimento anterior da perna e confere sensibilidade ao primeiro espaço da teia interdigital. 1 No curso para o pescoço fibular, o NFC toma o caminho do túnel fibular, uma banda osteofibrosa na origem do músculo fibular longo, que é um local comum de aprisionamento. 4

Muitos riscos de compressão do NFC foram descritos na literatura: o comportamento de cruzar as pernas por muito tempo e agachamento, massas compressivas intra e extraneural, anestesia recente, cirurgia e internação prolongada, diabetes, e alterações metabólicas como desnutrição e deficiência de complexo de vitaminas, 4 um achado habitual em obesos, pois 20% a 30% deles têm alguns distúrbios nutricionais que, após a cirurgia, podem piorar. 5

A perda de peso tem sido mostrada como uma causa habitual da neuropatia do NFC. A frequência de complicação neurológica foi relatada em 1,3% a 4,6% dos casos em pacientes bariátricos. 6 Algumas causas patológicas foram descritas: perda da proteção do tecido subcutâneo, o que ocasiona a compressão do nervo contra a estrutura dura do osso, um mecanismo imunológico de infiltração inflamatória na microestrutura do nervo, como uma caquexia, e as deficiências nutricionais. 7

Ao contrário, em pacientes que são atletas, o NFC é o mais afetado por lesões traumáticas, sendo o trauma contundente o mecanismo mais comum. Por outro lado, lesões de tração, embora menos comuns, poderiam ser mais graves. 8 Poucos esportes estão associados à paralisia não traumática do NFC, como síndrome compartimental crônica induzida por exercício. Peck et al., 9 em um estudo com pacientes corredores, relataram que um movimento repetitivo de flexão plantar e inversão combinadas em corridas ladeira abaixo ou em superfícies irregulares poderia causar um estiramento na cabeça fibular do NFC. No nosso caso, descrevemos um ciclista amador com uma lesão de NFC que pode ter piorado após perda de peso extrema e exercício de perna extenuante, que foi uma apresentação incomum de paralisia de NFC. Exercícios extenuantes podem causar microtraumas na barriga muscular, o que gera edema local e hematoma pequeno, que podem ser outras causas de compressão por exercício físico. 8

O tratamento é baseado na causa e na gravidade da paralisia nervosa. Quando os sintomas são intermitentes e é possível mudar o estilo de vida, um tratamento conservador deve ser feito. Mudanças de comportamento com relação ao posicionamento das pernas, ao sono, aos exercícios e à postura, proteção da cabeça fibular proeminente e reabilitação para alongamento dos grupos musculares contralaterais, e fortalecimento progressivo dos dorsiflexores são algumas práticas que devem ser realizadas. Quando qualquer lesão traumática ou uma função motora ou sensorial completa é perdida, ou não há qualquer sinal de melhora entre 6 semanas e 3 meses do tratamento conservador, uma abordagem cirúrgica e a microneurólise do NFC são indicadas. Bons resultados foram relatados após a cirurgia de neurólise, com 3% de algum déficit motor ou sensível. 2 9 10

A paralisia de NFC tem inúmeras causas, traumáticas e não traumáticas. No entanto, a perda de peso seguida de cirurgia bariátrica e a prática extenuante de esportes podem ser uma possível etiologia da paralisia de NFC.
